# Overexpression of a rice BAHD acyltransferase gene in switchgrass (*Panicum virgatum* L.) enhances saccharification

**DOI:** 10.1186/s12896-018-0464-8

**Published:** 2018-09-04

**Authors:** Guotian Li, Kyle C. Jones, Aymerick Eudes, Venkataramana R. Pidatala, Jian Sun, Feng Xu, Chengcheng Zhang, Tong Wei, Rashmi Jain, Devon Birdseye, Patrick E. Canlas, Edward E. K. Baidoo, Phat Q. Duong, Manoj K. Sharma, Seema Singh, Deling Ruan, Jay D. Keasling, Jenny C. Mortimer, Dominique Loqué, Laura E. Bartley, Henrik V. Scheller, Pamela C. Ronald

**Affiliations:** 1 Joint BioEnergy Institute, Lawrence Berkeley National Laboratory, Berkeley, CA 94720 USA; 2 Department of Plant Pathology and the Genome Center, University of California, Davis, CA 95616 USA; 3 Biomass Science and Conversion Technology Department, Sandia National Laboratories, CA94551, Livermore, USA; 4 Department of Microbiology and Plant Biology, University of Oklahoma, Norman, OK 73019 USA; 5 School of Biotechnology, Jawaharlal Nehru University, New Delhi, India; 6 Department of Bioengineering and Department of Chemical & Biomolecular Engineering, University of California, Berkeley, CA 94720 USA; 7 Department of Plant and Microbial Biology, University of California, Berkeley, CA 94720 USA

**Keywords:** Switchgrass, Bioenergy, Biofuel, Recalcitrance, Acyltransferase, *OsAT10*, Saccharification, Ferulic acid, *p*-coumaric acid

## Abstract

**Background:**

Switchgrass (*Panicum virgatum* L.) is a promising bioenergy feedstock because it can be grown on marginal land and produces abundant biomass. Recalcitrance of the lignocellulosic components of the switchgrass cell wall to enzymatic degradation into simple sugars impedes efficient biofuel production. We previously demonstrated that overexpression of *OsAT10,* a BAHD acyltransferase gene, enhances saccharification efficiency in rice.

**Results:**

Here we show that overexpression of the rice *OsAT10* gene in switchgrass decreased the levels of cell wall-bound ferulic acid (FA) in green leaf tissues and to a lesser extent in senesced tissues, and significantly increased levels of cell wall-bound *p*-coumaric acid (*p*-CA) in green leaves but decreased its level in senesced tissues of the T_0_ plants under greenhouse conditions. The engineered switchgrass lines exhibit an approximate 40% increase in saccharification efficiency in green tissues and a 30% increase in senesced tissues.

**Conclusion:**

Our study demonstrates that overexpression of *OsAT10*, a rice BAHD acyltransferase gene, enhances saccharification of lignocellulosic biomass in switchgrass.

**Electronic supplementary material:**

The online version of this article (10.1186/s12896-018-0464-8) contains supplementary material, which is available to authorized users.

## Background

Bioenergy derived from plant biomass is a renewable energy resource widely used for heat, electricity and transportation fuels [1]. Biofuels have the potential to reduce greenhouse gas emissions by reducing the use of fossil fuels [2]. First generation biofuels (i.e. biodiesel, bioethanol, and biogas) are mainly derived from starch, sugar and vegetable oil. Because these biofuels are produced from food or feed crops, less land is available to produce food, raising concerns about the effects of biofuels on food security. In contrast, second generation biofuels are derived from lignocellulosic feedstocks [3], most of which are non-food grasses such as *Panicum virgatum* (switchgrass) and *Miscanthus* x *giganteus*, woody plants such as *Populus trichocarpa* (poplar) and *Eucalyptus grandis* (eucalyptus), or crop residues such as corn stover [3].

Switchgrass, a fast-growing perennial C4 grass native to the United States, is considered a prime lignocellulosic feedstock for production of second generation biofuels [4]. Switchgrass is highly tolerant to water-deficit conditions and can be grown at large scale on marginal land. Switchgrass produces abundant lignocellulose, however, this biomass is not easily converted to simple sugars. This recalcitrance is due mainly to the structure of lignocellulose, including cellulose crystallinity, lignin, and lignin-carbohydrate complexes [5]. Knowledge of the genetic basis of cell wall composition and biosynthesis in switchgrass can help advance strategies to improve switchgrass as a bioenergy source.

Diverse approaches have been used to reduce the recalcitrance of plant cell walls. For example, researchers have reduced lignin content, altered lignin composition, structure and distribution in the cell wall [6–8], reduced inhibitors derived from biomass [9], increased the abundance of easily fermentable sugars [10, 11], and manipulated other components of the plant cell wall such as hemicellulose and pectin [12–14]. Genes controlling lignin biosynthesis that have been used in switchgrass engineering include 4-coumarate: coenzymeA ligase (4CL) [15], cinnamyl alcohol dehydrogenase (CAD) [16], and caffeic acid O-methyltransferase (COMT) [17–19]. Transcription factors related to cell wall biosynthesis and modification have also been used to engineer switchgrass, including a WRKY family transcription factor [20], an ERF gene (*PvERF001*) [21], a Knotted1-like gene *PvKN1* [22], two SBP-box-like genes *PvSPL1/2* [23], and a *PvMYB4* gene that encodes a general transcriptional repressor of the phenylpropanoid/lignin biosynthesis pathway [24, 25]. Researchers have also engineered switchgrass using microRNAs including miR156/*Corngrass1* (*Cg1*) to improve saccharification efficiency [26, 27].

BAHD acyltransferases utilize CoA thioesters and transfer an acyl group to oxygen and nitrogen molecules of diverse plant metabolites, and are important enzymes in cell wall modifications in grasses [28]. The ‘Mitchell clade’ of BAHD acyltransferases (ATs) is characterized by an expansion in the number of genes and by high gene expression in grasses relative to dicots [29]. Members of the Mitchell clade have been shown to play critical roles in biomass accumulation, cell wall polymer modification, and hydrolysis of lignocellulosic materials in grasses and are thus good candidates in feedstock engineering [28, 30–32]. In rice, overexpression of *OsAT1*0 has been used to enhance saccharification [30]. OsAT10 is a putative *p*-coumaroyl coenzyme A transferase that modifies glucuronoarabinoxylan (GAX) by altering the content of ferulic acid (FA) and *p*-coumaric acid (*p*-CA). FA and *p*-CA are phenylpropanoid-derived hydroxycinnamates (HCAs) that are a distinguishing feature of grass cell walls and other recently evolved commelinid monocots. FA is predominantly esterified to arabinose moieties on GAX in grasses [30] and *p*-CA is predominantly esterified to lignin [33]. However, FA has also been found to be incorporated into lignin in comelinids and other species, and *p*-CA also links to GAX [34, 35]. Feruloyl esters undergo oxidative coupling with neighboring phenylpropanoids on GAX and cause lignin polymerization, which strengthens both primary and secondary cell walls and increases recalcitrance [36]. Overexpression of *OsAT10* in rice increases the content of cell wall-bound *p*-CA and reduces the content of cell wall-bound FA. Biomass extracted from the *OsAT10* overexpression lines displayed up to a 40% increase in saccharification efficiency. Introduction of the *p*-CA monolignol transferases (PMTs) of rice or Brachypodium, another cell wall BAHD acyltransferase, into Arabidopsis and poplar promotes *para*-coumarylation of lignin and also improves saccharification [31, 37, 38]. These examples demonstrate the importance of the BAHD acyltransferases in modifying cell walls of grasses as well as dicots and illustrate their potentials in feedstock engineering to improve saccharification.

While the positive effect of increasing endogenous *OsAT10* expression in the model grass species rice has been studied, it has not been determined if the same approach can be used to improve saccharification in bioenergy grass species. In this study, we overexpressed the rice BAHD acyltransferase gene *OsAT10* in switchgrass. One of the two switchgrass lines overexpressing *OsAT10* showed no change in plant growth and development. Overexpression of the *OsAT10* gene in switchgrass significantly increased in the level of cell wall-bound *p*-CA in green leaves but decreased its level in senesced tissues, whereas the levels of cell wall-bound FA are decreased in green leaves and to a lesser extent in senesced tissues. The engineered switchgrass lines display 40% and 30% enhanced saccharification efficiency in green and senesced tissues, respectively. Thus, the *OsAT10* gene is a valuable target for improving biofuel production in bioenergy feedstocks.

## Results

### The OsAT10 orthologs are conserved in grasses

**Fig. 1:**
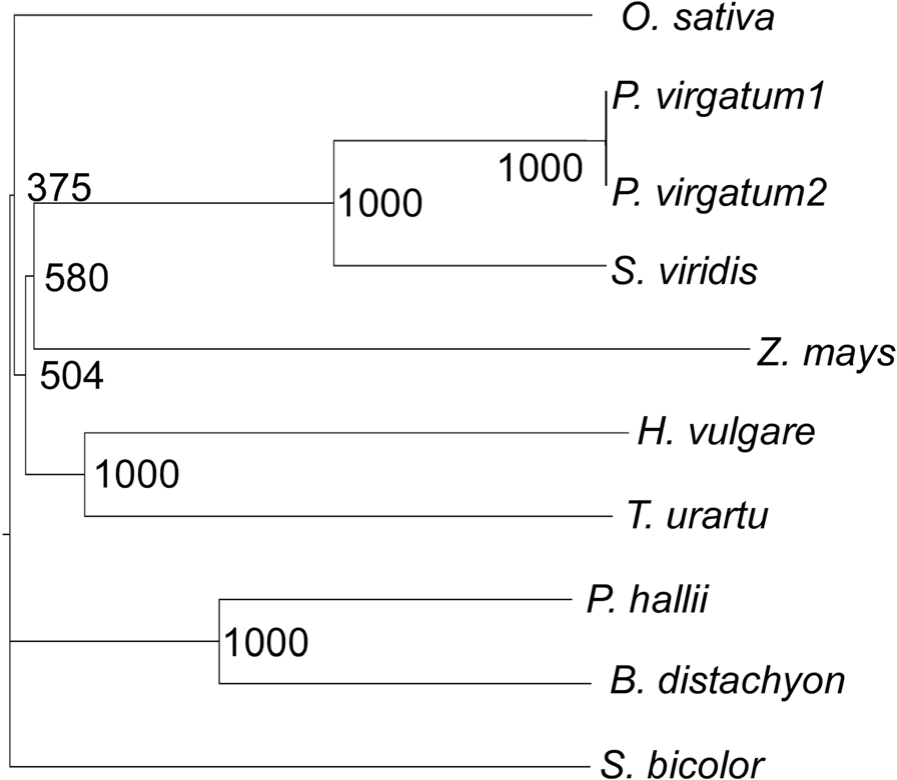
Phylogenetic tree of OsAT10 orthologs from selected grasses. The predicted amino acid sequences of OsAT10 orthologs from *Oryza sativa* (XP_015641801.1), *Setaria viridis* (Sevir.4G238000.1), *Panicum virgatum* (Pavir.J252500.1 and Pavir.Da00636.1), *Zea mays* (XP_008647983.1), *Hordeum vulgare* (BAK07645.1), *Triticum urartu* (EMS54808.1), *Panicum hallii* (Pahal.D00987.1), *Brachypodium distachyon* (XP_003563687.1), and *Sorghum bicolor* (XP_002438629.1) were aligned with the ClustalX2.1 program. Two putative OsAT10 orthologs were identified from switchgrass (*P. virgatum*), and Pavir.J252500.1 (81% identity to OsAT10) was designated as PvAT10 in this study based on its higher percentage of identity to OsAT10 and a higher expression level, compared to Pavir.Da00636.1 (79% identity to OsAT10). The phylogenetic tree was generated using the neighbor-joining (NJ) method with 1000 bootstrap values and was visualized using the FigTree program

To facilitate the transfer of the *OsAT10* engineering approach to other putative bioenergy crops, we analyzed *OsAT10* orthologs from multiple plant species, including switchgrass, sorghum and maize (Fig. 1). All selected grass species have one OsAT10 ortholog except switchgrass, which has two. The highly-expressed switchgrass orthologs (Pavir.J252500.1) is named as PvAT10 (Additional file 1: Figure S1). PvAT10 and OsAT10 share an 81% identity in their protein sequences (Additional file 2: Figure S2). PvAT10 is highly similar to its putative orthologs in other grass species as well, including *Panicum hallii* (91% identity), *Setaria viridis* (91% identity), *Sorghum bicolor* (84% identity) and *Brachypodium distachyon* (79% identity). The homolog confidence in *P. trichocarpa* and *E. grandis* is low (37% identity), consistent with OsAT10 being commelinid-specific. The PvAT10 and its homologs from other grass species contain a conserved transferase domain. PvAT10 and OsAT10 share an 86% identity in the transferase domain (Additional file 2: Figure S2), suggesting that these two encoded proteins should share similar functions.

### Overexpression of *OsAT10* in switchgrass

**Fig. 2:**
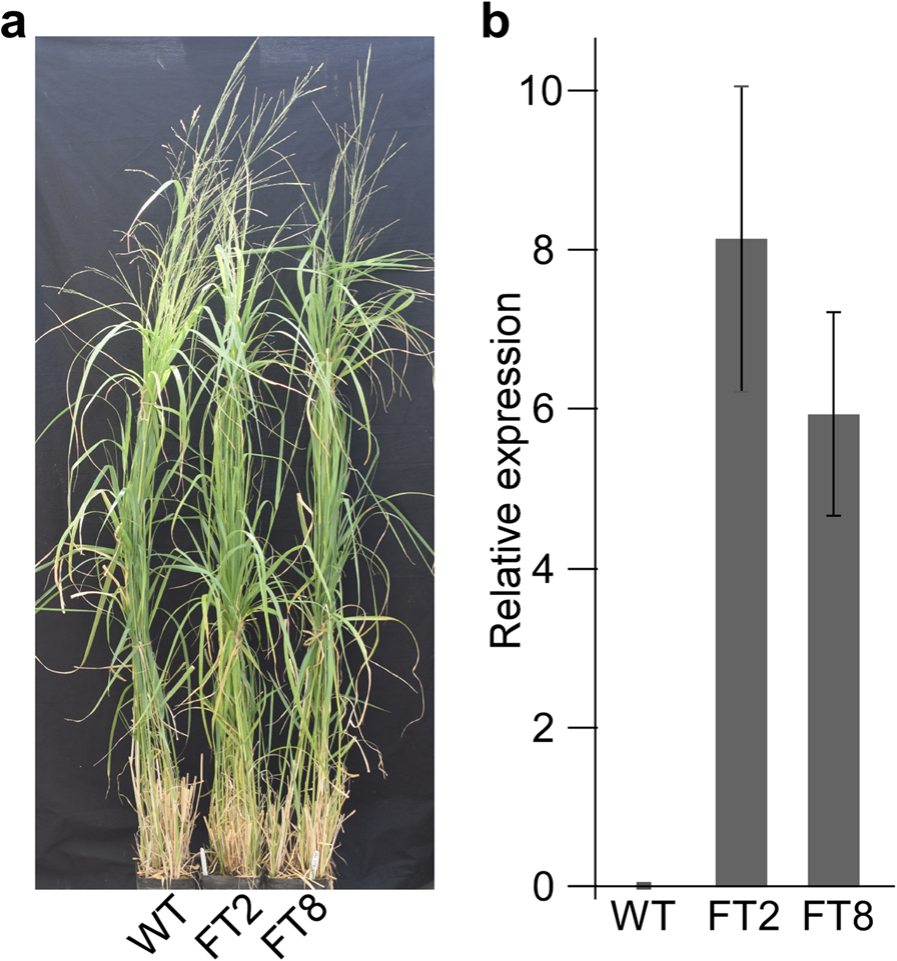
Switchgrass lines overexpressing *OsAT10*. **a** An image of transgenic switchgrass lines overexpressing *OsAT10* taken when plants were flowering. WT is the wild-type line, ‘Alamo’. FT2 and FT8 are two independent switchgrass transgenic lines overexpressing *OsAT10* in ‘Alamo’. **b** qRT-PCR assays of *OsAT10* in transgenic switchgrass lines. The ubiquitin gene was used as the internal control. Mean and standard deviation were calculated with data from three biological replicates for the wild-type line, and four biological replicates for each of the FT2 and FT8 lines. Three technical replicates were used for each biological replicate

To overexpress *OsAT10* in switchgrass, we transformed the wild-type switchgrass line ‘Alamo’ with a construct expressing the *OsAT10* gene driven by the maize *Ubi1* promoter [30]. We generated two independent transformed lines that possess the transgene (Additional file 3: Figure S3). Quantitative RT-PCR of green leaves revealed that lines FT2 and FT8 express *OsAT10* approximately 8- and 6-fold higher, respectively, than the reference ubiquitin gene, while the wild-type plants show no expression of *OsAT10* (Fig. 2). Line FT8 displays normal growth and development (Fig. 2); line FT2 is slightly shorter than the wild-type line and shows a slight decrease (14%) of biomass yield (Table 1).

**Table 1:** Height and dry weight of the mature wild-type and *OsAT10* overexpression switchgrass lines

Plant line	Height (cm) (mean ± SD)	Dry weight (g) (mean ± SD)	N^a^
WT^b^	201 ± 13	6.6 ± 1.7	20
FT2	174 ± 17^c^	4.8 ± 1.3^c^	20
FT8	201 ± 18	5.9 ± 1.9	20

^a^The number of tillers used for each line. Four biological replicates were used for each line and five tillers per replicate were used in the measurement

^b^WT is the wild-type line. FT2 and FT8 are two independently switchgrass transformant lines overexpressing *OsAT10*

^c^Asterisks indicate significant differences using the unpaired Student’s *t*-test (*P* < 0.01)

### The *OsAt10* overexpression lines display alterations in cell wall-bound phenolics in green leaves and to a lesser extent in senesced tissues

**Fig. 3:**
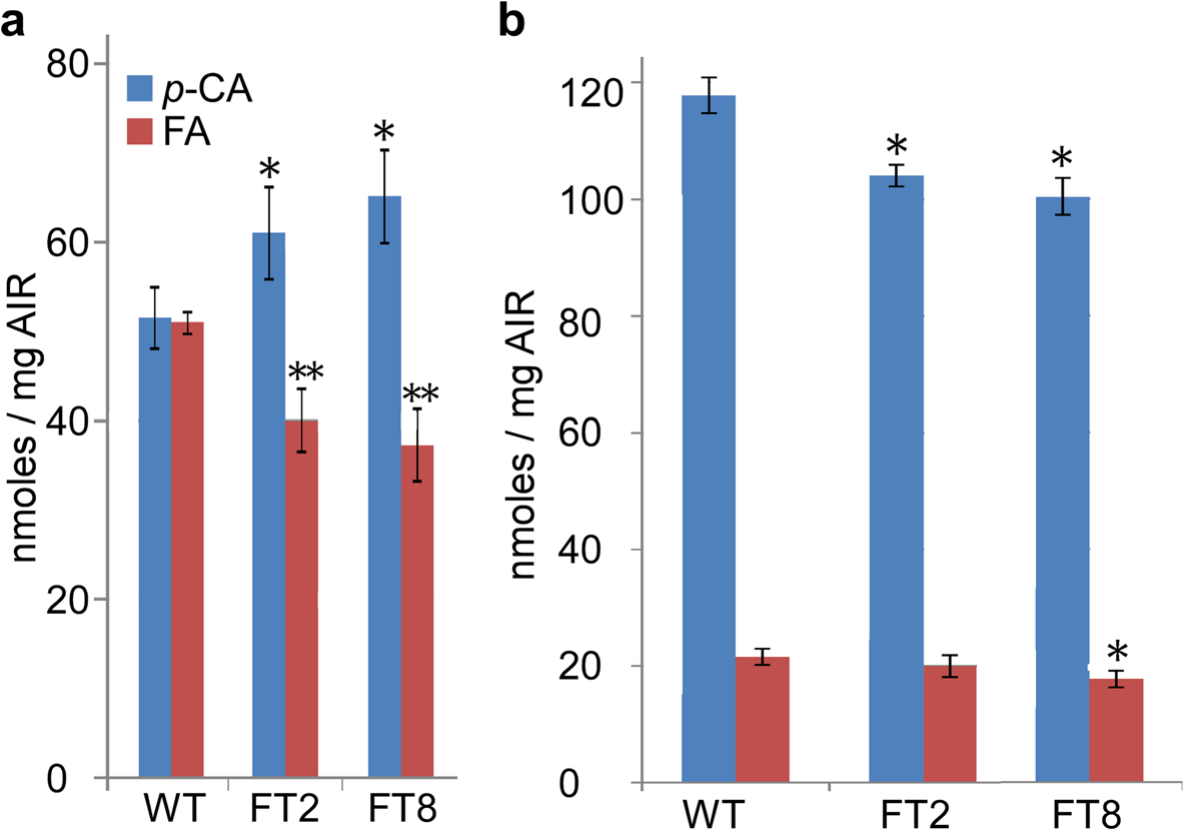
Switchgrass lines overexpressing *OsAT10* show alterations in cell wall-bound phenolics. **a** Quantitative analysis of cell wall-bound phenolics of green leaves of the wild-type (WT) (3 biological replicates) and *OsAT10* overexpressing switchgrass lines, FT2 (4 biological replicates) and FT8 (4 biological replicates). **b** Quantitative analysis of cell wall-bound phenolics from senesced tissues of the same set of switchgrass lines. The numbers of biological replicates of senesced tissues of lines WT, FT2 and FT8 are 4, 5, and 5, respectively. No technical replicates were used. Bars indicate standard deviation, and asterisks indicate significant differences using the unpaired Student’s *t*-test (*P < 0.05; **P < 0.01)

The grass cell wall is rich in hydroxycinnamic acids that affect cell wall recalcitrance [36, 39]. We evaluated the amount of FA and *p*-CA in green leaves of transgenic plants. Levels of cell wall-bound FA and *p*-CA are altered in alcohol insoluble residue (AIR) samples from green leaves (Fig. 3a and Additional file 4: Figure S4). The transgenic plants show higher levels of *p*-CA, an 18% increase in FT2 and a 28% increase in FT8. However, levels of FA are reduced in green leaves of transgenic plants- a 21% decrease in FT2 and a 27% decrease in FT8- compared with the wild-type control line (Fig. 3a). In senesced tissues of lines FT2 and FT8, the approximate 15% decrease in *p*-CA content is statistically significant (*p* < 0.05) compared with the wild type control. For FA, a statistically significant difference was observed in line FT8 (19% lower) but not in line FT2 (Fig. 3b).

### Overexpression of *OsAT10* does not alter the xylan sugar substitution pattern in switchgrass

**Fig. 4:**
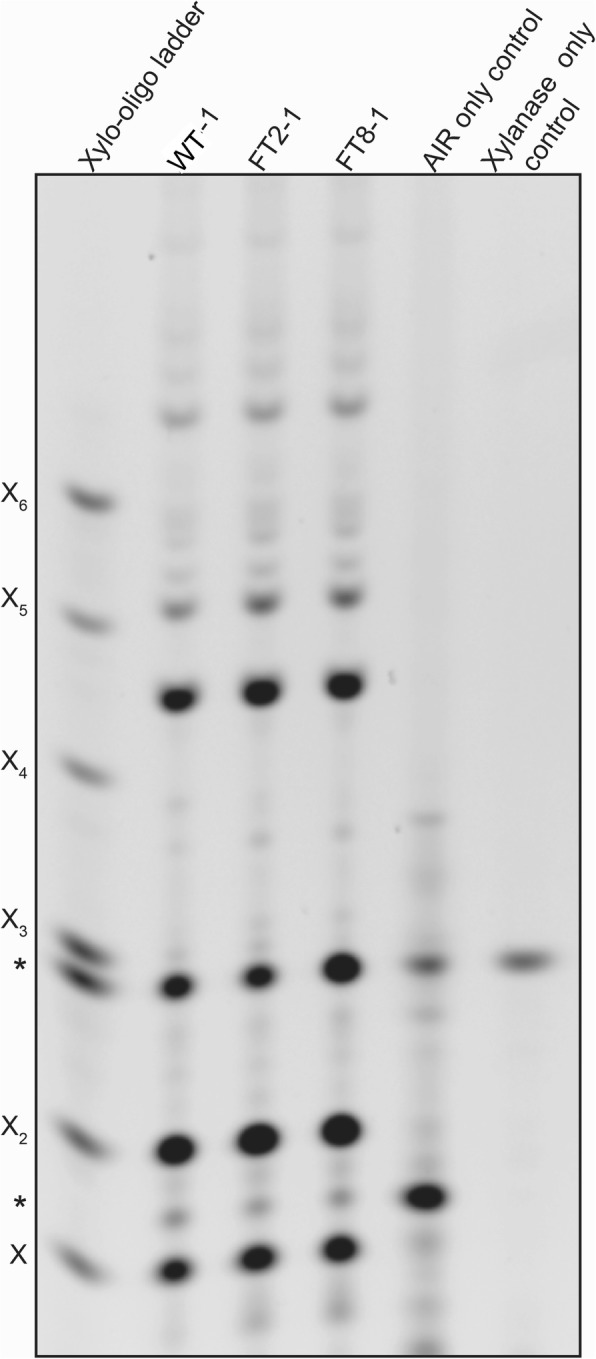
PACE fingerprint of xylanase GH11-digested AIR from the wild-type and engineered switchgrass lines. WT is the wild-type line. FT2 and FT8 are two independently transformed lines overexpressing *OsAT10*. Controls of AIR only (everything except the xylanase) and xylanase only (everything except the AIR) are included to identify background bands (marked with *). Xylo-oligo ladder: 20 pmol of xylose (X_1_) – xylohexaose (X_6_). Representative gel of three independent biological replicates of each line is shown

FA and *p*-CA are important in cell wall polymer modification and can be ester linked to xylan [30, 40]. We used polysaccharide analysis by carbohydrate gel electrophoresis (PACE) to determine the xylan structure of the *OsAT10* overexpression switchgrass [41]. For this analysis, AIR samples of two-week-old leaves were prepared and digested with a GH11 xylanase. The digested samples were used to run the PACE gel. Compared to the wild-type line, *OsAT10* overexpression lines FT2 and FT8 do not show significant differences in the pattern of xylanase-released oligosaccharides (Fig. 4). The number of fragments and the intensity of each band for lines FT2 and FT8 are similar to those of the wild-type line, indicating that overexpression of *OsAT10* had no obvious impact on the xylan sugar substitution pattern in switchgrass.

### Overexpression of *OsAT10* results in enhanced saccharification

**Fig. 5:**
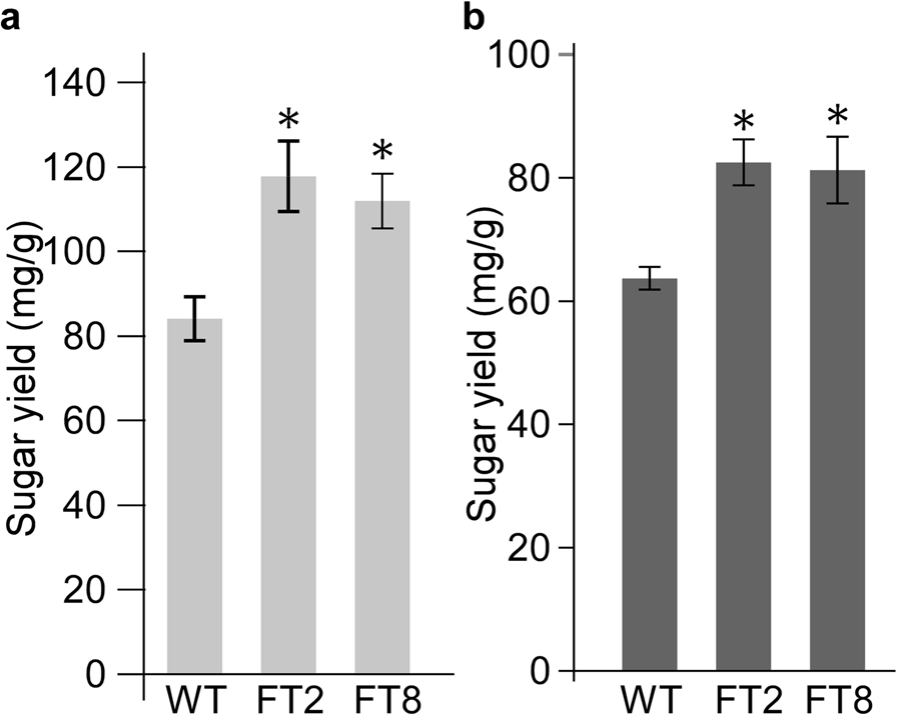
Saccharification assays of green leaves (**a**) and senesced tissues (**b**) of the wild-type (WT) and *OsAT10* overexpression switchgrass lines (FT2 and FT8). Reducing sugars released from biomass were measured using the 3,5-dinitrosalicylic acid (DNS) method. Bars represent mean ± standard deviation. The numbers of biological replicates for green leaf tissues of lines WT, FT2 and FT8 are 3, 4 and 4, respectively, and 4, 5, and 5, respectively, for senesced tissues. Asterisks indicate significant differences using the unpaired Student’s *t*-test (*P < 0.05)

To evaluate the effect of overexpression of *OsAT10* on cell wall recalcitrance in switchgrass, we conducted saccharification assays on green leaves and senesced tissues using a hot water pretreatment. Enhanced saccharification was observed in both green leaves and senesced tissues (Fig. 5). In green leaf tissues, lines FT2 and FT8 released 40% and 33% more reducing sugars, respectively, compared to the wild-type line (Fig. 5a). Similarly, approximately 30% more reducing sugars were released from senesced tissues of FT2 and FT8 than the wild-type line (Fig. 5b). These data demonstrate that biomass from the *OsAT10* overexpression lines are less recalcitrant to enzymatic digestion, leading to higher sugar yields after saccharification.

## Discussion

In this study, we show that overexpression of a rice BAHD acyltransferase, *OsAT10*, in switchgrass reduces recalcitrance and enhances biomass saccharification efficiency, demonstrating that *OsAT10* functions similarly in rice and switchgrass and that the Os*AT10* engineering approach is feasible in switchgrass and possibly in a wide variety of other bioenergy feedstocks as well. Moreover, overexpression of *OsAT10* has no or marginal effects on plant growth and development, similar to the observation in the *OsAT10* overexpression rice lines [30]. One of the *OsAT10* overexpression switchgrass lines (FT8) displays normal plant growth and line FT2 is slightly reduced in biomass yield. The reduced yield observed in line FT2 may be due to somatic variations induced during the transformation process, but not the transgene per se. Nonetheless, the negative effect of the *OsAT10* overexpression, if any, is manageable, which indicates the feasible application of *OsAT10* to other biofuel feedstocks.

Both the *OsAT10* overexpression rice and switchgrass lines show enhanced saccharification. However, different from the rice *OsAT10* lines that show a 20% increase in cell wall glucose content, the cell wall composition of the *OsAT10* overexpression switchgrass lines is the same as the wild-type line (Additional files 5: Table S1 and Additional file 6: Table S2), indicating that the glucose content is not the major reason for enhanced saccahrification of the engineered switchgrass lines.

The enhanced saccharification of the *OsAT10* switchgrass lines is negatively correlated with cell wall-bound FA, consistent with the findings in rice [30] and a previous study in which overexpression of a transcription factor *PvMYB4* reduces cell wall-bound phenolics and enhances saccharification [25]. Different from that observed in the *OsAT10* overexpression rice lines in which the levels of *p*-CA are increased both in the young seedlings and mature plant biomass, the level of *p*-CA is increased in green leaf tissues but decreased in senesced tissues in the *OsAT10* switchgrass lines. However, in all the rice and switchgrass tested, the level of FA is reduced compared to the control lines. Taken all together, cell wall-bound FA rather than *p*-CA should be the major factor of the biomass recalcitrance.

The improved enzyme accessibility due to the reduced level of cell wall-bound FA could be the major reason for enhanced saccharification of the *OsAT10* switchgrass lines. Cell wall-bound FA is an important component of recalcitrance because FA typically forms linkages between cell wall polymers [40]. The reduced level of cell wall-bound FA in the engineered switchgrass lines reduces the association between lignin and polysaccharides such as arabinoxylans, and in turn improves saccharification. Cell wall-bound FA as an important component of recalcitrance is also shown in studies using feruloyl esterases. The feruloyl esterase cleaves the ester linkage between hydroxycinnamoyl substrates and polysaccharides, which enhances the access of hydrolases to the polysaccharide. When added to the enzyme cocktail, feruloyl esterase shows synergy with cellulases, xylanases, pectinases, and accessory enzymes in degrading cell walls and enhancing saccharification [40]. Compromised plant cell wall integrity and disease resistance is associated with decreased levels of polysaccharide feruloylation or cell wall phenolics in several plant species [42–44], indicating the importance of cell wall phenolics in maintaining the proper cell wall structure, biotic stress resistance, and recalcitrance. FA and *p*-CA can be ester linked to xylan [30, 40]. While overexpression of *OsAT10* clearly alters the FA and *p*-CA content in xylan, assays in the current study are not sufficient to explain the mechanism. Moreover, one puzzle is that the BAHD acyltransferases are cytoplasmic but esterification of xylan takes place inside the Golgi [45]. Based on these results, it has been hypothesized that acyltransferases such as OsAT10 transfer the FA or *p*-CA to a cytoplasmic intermediate, for example a protein or UDP-arabinofuranose [40, 46], but so far such activity has not been demonstrated and needs to be studied in the future.

## Conclusion

Overexpression of the rice BAHD acyltransferase gene *OsAT10* alters the levels of cell wall-bound FA and *p*-CA and enhances saccharification with no or minimal negative effects on plant growth, suggesting that this approach may be useful for future engineering of bioenergy feedstocks.

## Methods

### Plant materials

The lowland-type switchgrass (*Panicum virgatum L.*) cultivar ‘Alamo’ [47] was used in this study. Switchgrass plants were grown and propagated in the greenhouse at the University of California, Davis. The light intensity was maintained at approximately 250 μmol m^− 2^ s^− 1^ with the supplement of artificial lights during the winter time and the day/night period was set to 14/10 h. The temperature was ~ 28–30 °C. In this study, biological replicates of the same line were clonally propagated from a single plant and were grown in different pots. When plants were fully senesced (tillers that senesce and become dry after flowering), the R4 stage [48], 20 tillers from each line, that is, five top tillers per plant and four plants per line, were measured for plant height and dry weight.

### Phylogenetic analysis of the OsAT10 orthologs

The predicted protein sequence of *OsAT10* (LOC_Os06g39390) [49] was used to search the protein databases of *Arabidopsis thaliana*, *Brachypodium distachyon*, *Eucalyptus grandis*, *Glycine max*, *Hordeum vulgare*, *Panicum hallii*, *Panicum virgatum*, *Populus trichocarpa, Setaria viridis*, *Sorghum bicolor, Triticum urartu*, and *Zea mays* at Phytozome (http://jgi.doe.gov/) or Ensembl Plants (http://plants.ensembl.org/index.html) [50, 51]. The identified protein sequences were aligned with the ClustalX2.1 program and the phylogenetic tree was generated using the neighbor-joining (NJ) method with 1000 bootstraps and was visualized using FigTree 1.4.2. The transferase domain of OsAT10 orthologs was identified using the SMART program [52] and aligned with the ClustalX2.1 program [53].

### Generation of *Ubipro*: *OsAT10* switchgrass lines

The binary vector containing the rice *OsAT10* gene under control of the maize *Ubiquitin* promoter was described in a previous study [30]. For switchgrass transformation, fresh calli were induced from inflorescences of switchgrass and transformed with the *OsAT10* construct using *Agrobacterium*-mediated transformation as described [54]. Regenerated plantlets were rooted in a mixture containing equal volumes of liquid MP media, topsoil, and Sunshine Mix. Hygromycin was used as the selection antibiotic to select putative transgenic lines. Positive transformants were identified using regular PCR with *OsAT10*-specific primers FT/1F (5′-cgtcgagccgcacaacag-3′) and FT/1R (5′-gatgaggtcgca gttcacca-3′). The positive transgenic lines were then transferred to greenhouses under the conditions described above. Genomic DNA was isolated from young leaves using the CTAB method [55]. The ubiquiin gene was used as the DNA quality control and the primers were Ubi/QF (5′-agaagcgcaagaagaagacg-3′) and Ubi/QR (5′-ccaccttgtagaactggagca-3′). The PCR was conducted on the T100 thermal cycler (Bio-Rad) using the DreamTaq polymerase (Thermo Scientific).

### qRT-PCR

The expression level of the *OsAT10* gene in the transgenic switchgrass lines was analyzed using quantitative reverse transcription polymerase chain reaction (qRT-PCR). Green leaves were sampled with liquid nitrogen and stored in a − 80 °C freezer. Total RNAs were isolated with TRIzol (Invitrogen, CA) as described [56]. RNA was treated with DNase I (Ambion) to remove residual DNA. cDNA synthesis was conducted using the high-capacity cDNA reverse transcription kit (Applied BioSystems) following the manufacturer’s instructions. qRT-PCR was performed on a Bio-Rad CFX96 Real-Time System coupled to a C1000 Thermal Cycler (Bio-Rad). The Bio-Rad SsoFast EvaGreen Supermix was used in qRT-PCR reactions. The qRT-PCR condition was set as 40 cycles with denaturation at 95 °C for 5 s and annealing together with amplification at 60 °C for 30 s. The relative quantification of each transcript was calculated using the 2-^Δ^CT method, normalized to the internal control ubiquitin gene (accession number: FE609298). The results were fromthree biological replicates for the wild-type line, and four biological replicates for each of the FT2 and FT8 lines. Three technical replicates were used for each biological replicate. *OsAT10*-specific primers used in the qRT-PCR assays were FT/QF (5′-gtacgtgtcggactggagcaa-3′), FT/QR (5′-gacgatcaccgacgagatgag-3′). The ubiquitin-specific primers of switchgrass were Ubi/QF (5′-agaagcgcaagaagaagacg-3′) and Ubi/QR (5′-ccaccttgtagaactggagca-3′).

### Cell wall-bound phenolics extraction

Biomass of green leaf tissues was used to measure cell wall-bound phenolics. Green leaf tissues were collected and snap frozen in liquid nitrogen prior to being dried in the lyophilizer (Labconco) at − 80 °C overnight. Dried samples were homogenized with a sample mill and then were ground on a tissue homogenizer in 2-mL polypropylene tubes at 1200 rpm with one stainless-steel ball for four times, 90 s each time. Alcohol insoluble residue (AIR) preparation and the de-starch procedure was conducted as described [30]. Briefly, tissues were processed following instructions described above. Ground tissue (30 mg) was treated with 95% ethanol at 100 °C for 30 min. After the treatment, the supernatant was discarded by centrifugation (10,000 *g*, 8 min), and the residue was washed three times with 70% ethanol and dried at approximately 35 °C under vacuum. The dried powder obtained after 70% ethanol wash is designated as alcohol insoluble residues (AIR). The dried AIR sample was de-starched with amylase (Novozymes), amyloglucosidase (Megazyme) and pullulanase (Megazyme) as described [30]. De-starched AIR sample (10 mg) was mixed with 500 μL of 2 M NaOH and incubated at 1400 rpm at 30 °C for 24 h. Concentrated HCl (100 μL) was added to the mixture and then subjected to three ethyl acetate partitioning steps. Ethyl acetate fractions were pooled, dried and suspended in 50% methanol for the subsequent LC-MS analysis [9]. Senesced tissues were processed in the same procedure except being dried in the oven. The numbers of biological replicates of green leaf tissues of lines WT, FT2 and FT8 are 3, 4, and 4, respectively. The numbers of biological replicates of senesced tissues for lines WT, FT2 and FT8 are 4, 5, and 5, respectively. No technical replicates were used in the assays.

### Polysaccharide analysis by carbohydrate gel electrophoresis (PACE)

PACE was performed as described [41]. Briefly, 100 μg AIR of each sample was pre-treated with 4 M NaOH (20 μl) for 1 h at 21 °C. Following neutralization with 1 M HCl, each sample was suspended in a final concentration of 100 mM ammonium acetate, pH 6.0 (500 μl). Samples were hydrolyzed to completion with 10 μl (8.5 mg/mL) CAZy (www.cazy.org) GH11 family endo-β-1, 4-xylanase (Prozomix) from *Neocallimastix patricarium* (accession number: CAA46498). The released oligosaccharides, along with appropriate controls and xylo-oligosaccharide standards (Megazyme) were dried *in vacuuo*, and were reductively aminated with 8-aminonaphthalene-1, 3, 6-trisulfonic acid (ANTS; Invitrogen, www.life technologies.com) as follows: to each tube was added 5 μl of 0.2 M ANTS solution (resuspended in 17:3, water: acetic acid) and 5 μl of 0.2 M 2-picoline borane (Sigma, resuspended in DMSO). Samples were incubated overnight in the dark in a 37 °C waterbath, then dried *in vacuuo* and resuspended in 100 μl of 3 M urea. The samples (2 μl) were analyzed by separation on large format Tris-borate acrylamide gel prepared as described [57]. The PACE gels were visualized with a G-box (Syngene, www.syngene.com) equipped with a UV detection filter and long-wave UV tubes (365 nm emission). Experiments were performed on three biological replicates of the wild-type line and four biological replicates for each of the FT2 and FT8 lines and a representative gel was shown.

### Compositional analysis

Compositional analysis of switchgrass lines was conducted according to the NREL acidolysis protocol (LAP) LAP-002 [58, 59]. Fully senecesed tissues were used. Three biological replicates for each of the wild-type and FT8 lines and four biological replicates for line FT2 were used in the assay. Homogenized switchgrass biomass (100 mg) and 1 mL of 72% H_2_SO_4_ were incubated in a shaker at 300 rpm and 30 °C for 1 h. The sample was diluted to 4% H_2_SO_4_ by adding 28 mL distilled water and autoclaved at 121 °C for 1 h. After autoclaving, the sample was placed in ice to quench the reaction and was filtered. The filtrate was used to measure the carbohydrate concentration on an Agilent HPLC 1200 Series equipped with a Bio-Rad Aminex HPX-87H column and a Refractive Index detector. A 4 mM H_2_SO_4_ solution was used as the mobile phase in running the machine at the speed of 0.6 mL min^− 1^. The column temperature was set at 60 °C. The injection volume was 20 μL with a run time of 25 min. The solid part was heated at 105 °C overnight to measure the combined weight of lignin and ash.

### Monosaccharide composition analysis

Senesced switchgrass tissues, three biological replicates per line, were used in the monosaccharide composition analysis. De-starched AIR (10 mg) was hydrolyzed with 2 M Trifluoroacetic acid (TFA) at 121 °C for 1 h. The hydrolysate was dried in a CentriVap at 32 °C overnight and then dissolved in water. The dissolved hydrolysate was filtered through a 0.45 μm PVDF filter and the filtrate was analyzed by high-performance anion-exchange chromatography with pulsed amperometric detection (HPAEC-PAD) on a Dionex ICS-3000 system with the CarboPac PA20 column (3 × 150 mm) [60].

### Cell wall pretreatments and saccharification

Liquid hot water pretreatment was used in the saccharification assay [9]. Water (340 μL) was added to 10 mg de-starched AIR samples made from senesced tissues and green leaves as previously described [30]. The biomass mixture was shaken at 1400 rpm at 30 °C for 30 min and autoclaved at 120 °C for 1 h. In saccharification, pretreated biomass was hydrolyzed with 650 μL of 100 mM sodium citrate buffer pH 5 containing 80 μg/mL tetracycline and 1% *w*/w Cellic CTec2 enzyme mixture (Novozymes), which contains cellulases, β-glucosidases, and hemicellulases. After incubation at 50 °C with shaking (800 rpm) for 95 h, samples were centrifuged at 20000 g for 3 min and 10 μL of the supernatant was used for measurement of reducing sugars using the 3,5-dinitrosalicylic acid (DNS) assay [61]. The absorbance of the samples was measured in a spectrophotometer at 540 nm. Glucose solutions were used for the standard curve. The number of biological replicates and technical replicates for each line was the same as the one for the assay of cell wall-bound phenolics.

## Additional files

Additional file 1: **Figure S1.** Gene expression levels of the two *PvAT10* genes from different switchgrass tissues. (DOCX 68 kb)

Additional file 2: **Figure S2.** Sequence alignment of the acyl transferase domain from OsAT10 orthologs of selected grasses. (DOCX 629 kb)

Additional file 3: **Figure S3.** Analysis of the *OsAT10* transgene in switchgrass lines. (DOCX 33 kb)

Additional file 4: **Figure S4.** Representative LC-MS chromatograms of *p*-coumarate (a, b) and ferulate (c, d) obtained from a plant extract (a, c) and authentic standards (b, d). (DOCX 59 kb)

Additional file 5: **Table S1.** Compositional analysis of senesced tissues of the wild-type and *OsAT10* overexpression switchgrass lines. (DOCX 20 kb)

Additional file 6: **Table S2.** Cell wall monosaccharide composition of senesced switchgrass tissues. (DOCX 21 kb)

## Abbreviations

4CL: 4-Coumarate: coenzymeA ligase
AIR: Alcohol insoluble residue
AT: Acyltransferase
BAHD: The initials of the first four biochemically characterized enzymes in this class, benzylalcohol O-acetyltransferase (BEAT), anthocyanin O-hydroxycinnamoyltransferase (AHCT), anthranilate N-hydroxycinnamoyl/benzoyltransferase (HCBT) and deacetylvindoline 4-O-acetyltransferase (DAT)
CAD: Cinnamyl alcohol dehydrogenase
COMT: Caffeic acid O-methyltransferase
FA: Ferulic acid
GAX: Glucuronoarabinoxylans
MLG: Mixed-linkage glucan
PACE: Polysaccharide analysis by carbohydrate gel electrophoresis
*p*-CA: *P*-coumaric acid
qRT-PCR: Quantitative reverse transcription polymerase chain reaction
TFA: Trifluoroacetic acid
