# Supplementary material for: Overexpression of a rice BAHD acyltransferase gene in switchgrass (Panicum virgatum L.) enhances saccharification
Source: BMC Biotechnol. 2018 Sep 4;18:54. doi: 10.1186/s12896-018-0464-8 (PMC6123914; doi:10.1186/s12896-018-0464-8)
Supplement: Supplementary file 1 — Figure S1. Gene expression levels of the two PvAT10 genes from different switchgrass tissues. (DOCX 68 kb) [file 12896_2018_464_MOESM1_ESM.docx]

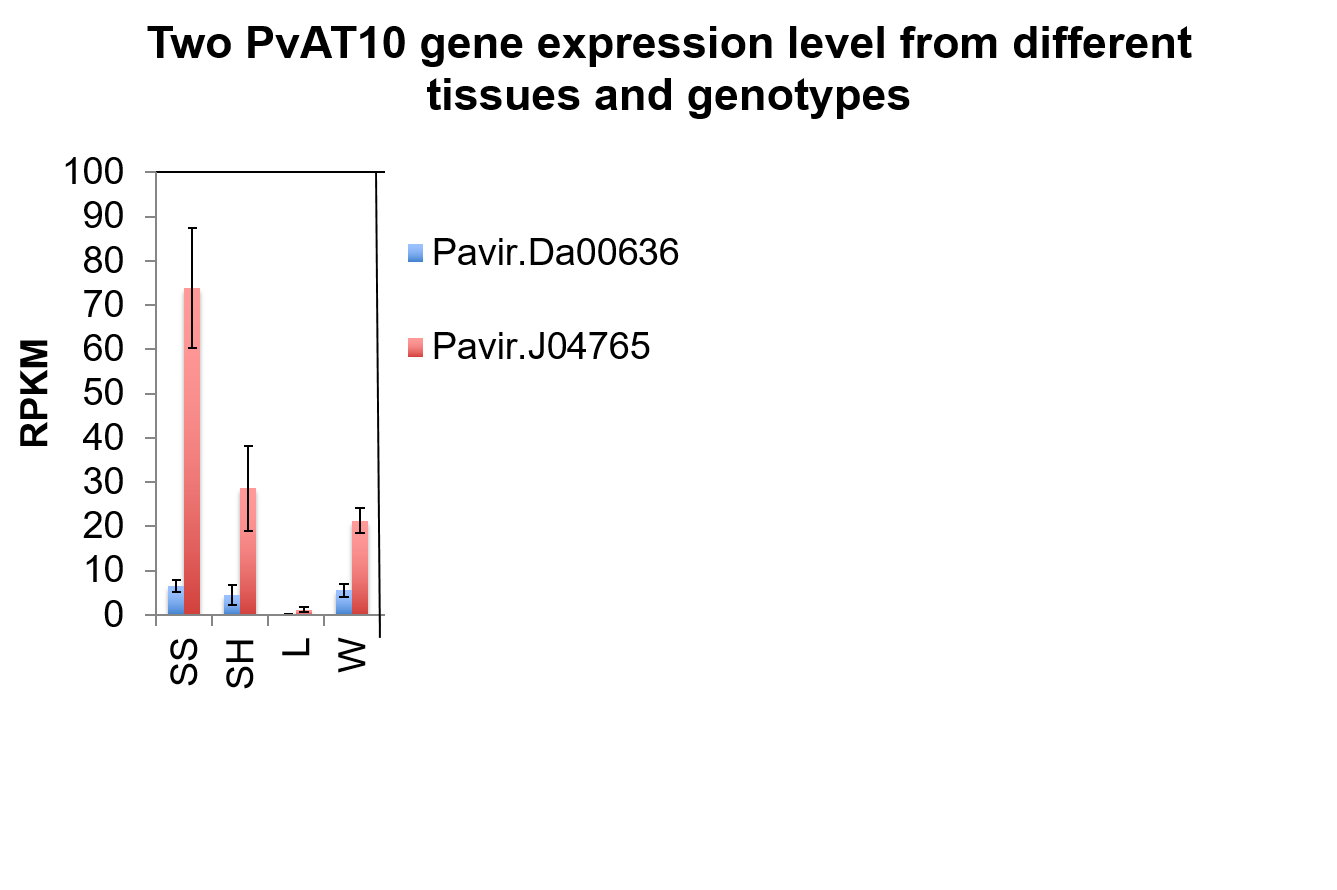


**Additional file 1: Fig. S1.** Gene expression levels of the two *PvAT10* genes from different switchgrass tissues. Pavir.J04765 is the gene ID for PvAT10 (Pavir.J252500.1) used in Figure 1. All samples are from the E4 stage. SS – lower part of 2^nd^ internode; SH - upper part of 2^nd^ internode; L- leaf with sheath from 2^nd^ internode; W- whole tiller. Error bars represent standard deviation of three replicates. RPKM: reads per kilobase per million mapped reads.
