# Supplementary material for: Overexpression of a rice BAHD acyltransferase gene in switchgrass (Panicum virgatum L.) enhances saccharification
Source: BMC Biotechnol. 2018 Sep 4;18:54. doi: 10.1186/s12896-018-0464-8 (PMC6123914; doi:10.1186/s12896-018-0464-8)
Supplement: Supplementary file 2 — Figure S2. Sequence alignment of the acyl transferase domain from OsAT10 orthologs of selected grasses. (DOCX 629 kb) [file 12896_2018_464_MOESM2_ESM.docx]

**

Additional file 2: Fig. S2.** Sequence alignment of the acyl transferase domain from OsAT10 orthologs of selected grasses. Predicted OsAT10 ortholog protein sequences from *Setaria viridis*, *Panicum virgatum*, *Panicum hallii*, *Sorghum bicolor, Zea mays*, *Oryza sativa*, *Hordeum vulgare*, *Triticum urartu*, and *Brachypodium distachyon* were aligned with the ClustalX2.1 program. Conserved amino acid residues are highlighted with asterisks.
