# Supplementary material for: Overexpression of a rice BAHD acyltransferase gene in switchgrass (Panicum virgatum L.) enhances saccharification
Source: BMC Biotechnol. 2018 Sep 4;18:54. doi: 10.1186/s12896-018-0464-8 (PMC6123914; doi:10.1186/s12896-018-0464-8)
Supplement: Supplementary file 3 — Figure S3. Analysis of the OsAT10 transgene in switchgrass lines. (DOCX 33 kb) [file 12896_2018_464_MOESM3_ESM.docx]

**Additional file 3: Fig. S3.** Analysis of the *OsAT10* transgene in switchgrass lines. *OsAT10*-specific primers were used to detect the *OsAT10* transgene and the ubiquitin gene was used as the control. WT is the nontransgenic wild-type line and FT2 and FT8 are two independently transformed lines.
