# Supplementary material for: Overexpression of a rice BAHD acyltransferase gene in switchgrass (Panicum virgatum L.) enhances saccharification
Source: BMC Biotechnol. 2018 Sep 4;18:54. doi: 10.1186/s12896-018-0464-8 (PMC6123914; doi:10.1186/s12896-018-0464-8)
Supplement: Supplementary file 4 — Figure S4. Representative LC-MS chromatograms of p-coumarate (a, b) and ferulate (c, d) obtained from a plant extract (a, c) and authentic standards (b, d). (DOCX 59 kb) [file 12896_2018_464_MOESM4_ESM.docx]

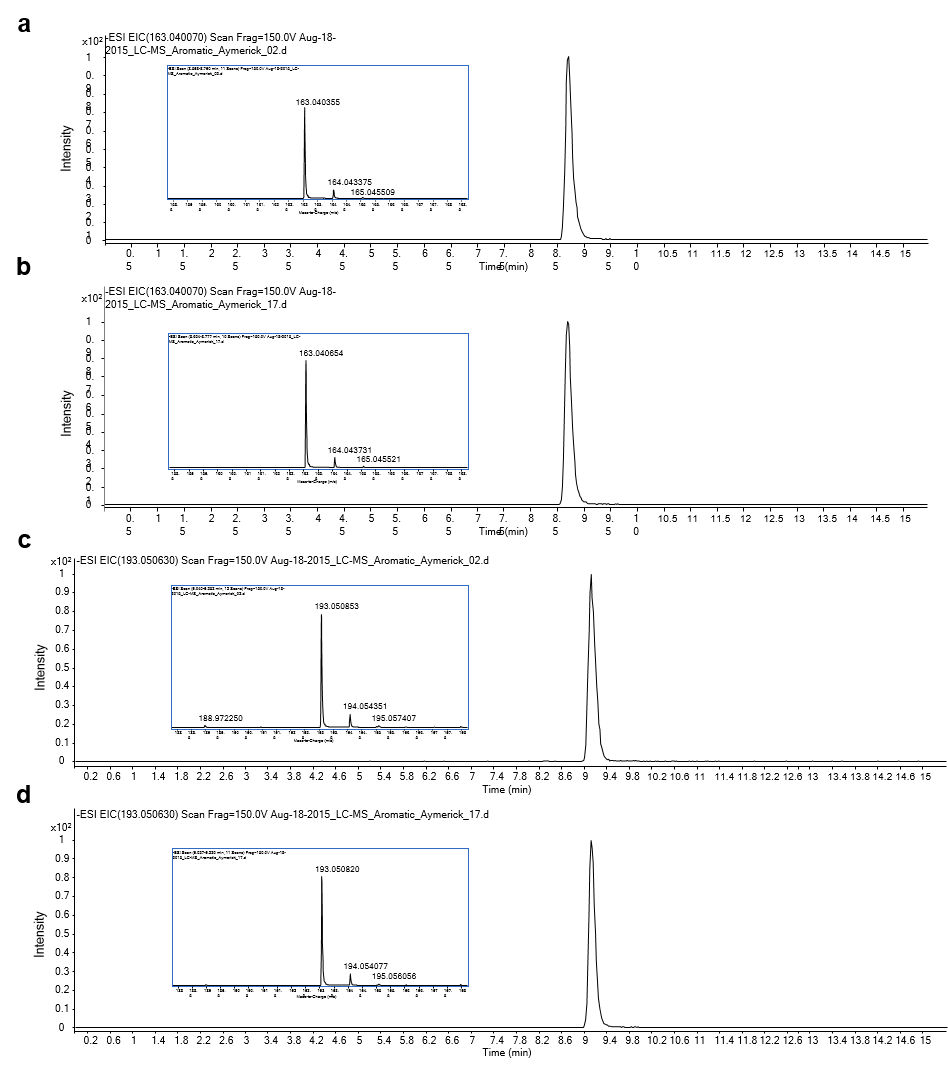


**Additional file 4: Fig. S4.** Representative LC-MS chromatograms of *p*-coumarate (a, b) and ferulate (c, d) obtained from a plant extract (a, c) and authentic standards (b, d).
