# Supplementary material for: Overexpression of a rice BAHD acyltransferase gene in switchgrass (Panicum virgatum L.) enhances saccharification
Source: BMC Biotechnol. 2018 Sep 4;18:54. doi: 10.1186/s12896-018-0464-8 (PMC6123914; doi:10.1186/s12896-018-0464-8)
Supplement: Supplementary file 5 — Table S1. Compositional analysis of senesced tissues of the wild-type and OsAT10 overexpression switchgrass lines. (DOCX 20 kb) [file 12896_2018_464_MOESM5_ESM.docx]

**Additional file 5: Table S1.** Compositional analysis of senesced tissues of the wild-type and *OsAT10* overexpression switchgrass lines

| Plant line | Composition of biomass (%) | | | |
| --- | --- | --- | --- | --- |
|  | Glucose | | Xylose | Lignin |
| WT | 33.4±3.0 | 18.7±1.1 | | 16.1±0.6 |
| FT2 | 34.2±2.5 | 20.1±1.3 | | 16.1±0.8 |
| FT8 | 35.5±1.3 | 19.9±1.2 | | 15.6±0.4 |

Composition was determined with the NREL protocol. WT is the wild-type line. FT2 and FT8 are two independently switchgrass transformant lines overexpressing *OsAT10*. Three biological replicates of lines WT and FT8, respectively, and four FT2 replicates were used in the assay. No significant differences were observed.
