# Supplementary material for: Overexpression of a rice BAHD acyltransferase gene in switchgrass (Panicum virgatum L.) enhances saccharification
Source: BMC Biotechnol. 2018 Sep 4;18:54. doi: 10.1186/s12896-018-0464-8 (PMC6123914; doi:10.1186/s12896-018-0464-8)
Supplement: Supplementary file 6 — Table S2. Cell wall monosaccharide composition of senesced switchgrass tissues. (DOCX 21 kb) [file 12896_2018_464_MOESM6_ESM.docx]

**Additional file 6: Table S2.** Cell wall monosaccharide composition of senesced switchgrass tissues (nmol/mg AIR)

|  | Rha | Ara | Gal | Glc | Xyl | GalA |
| --- | --- | --- | --- | --- | --- | --- |
| WT | 12±2 | 234±20 | 63±11 | 137±31 | 1015±13 | 31±5 |
| FT2 | 10±2 | 237±39 | 57±11 | 104±13 | 1021±83 | 31±8 |
| FT8 | 10±1 | 235±28 | 58±9 | 126±26 | 1068±54 | 31±7 |

AIR, alcohol insoluble residue; Rha, rhamnose; Ara, arabinose; Gal, galactose; Glc, glucose; Xyl, xylose; GlaA, galacturonic acid. The data of fucose and glucuronic acid are not shown here because their concentrations are too low to be detected accurately. WT indicates the wild-type line. FT2 and FT8 are two independently transformed lines overexpressing *OsAT10*. Three biological replicates were used for each line. Numbers are mean ± standard deviation, and no significant differences were detected.
